# French Psychological Literature
*Annales Medico-Psychologiques. Janvier, 1854.


**Published:** 1855-01-01

**Authors:** 


### Art. VI.—FRENCH PSYCHOLOGICAL LITERATURE.*

In a late number of this journal, we had occasion to show, that whatever merit there may be in having introduced to the notice of British medical practitioners the so-called "Non-restraint" system of treatment in mental disease, is undoubtedly due to Dr. Charlesworth, and not to Mr. Gardiner Hill, by whom the honour has been vociferously claimed. We, at the same time, endeavoured to name the true originator of the humane views which, at the present day, govern our management of lunatics. We claimed for Pinel all the glory of the revolution that has since his day transformed the lunatic asylum from a cage or a dungeon, to a drawing-room or library—a revolution that has opened the doors of the cells of incurables, and let in the messengers of mercy carrying health and sanity in their hands—a revolution that has, within less than half a century, left nought but historic trace of the horrors and cruelties which were the ordinary fate of the inmates of asylums. And we thought we had but done honour to whom honour was due; the article, however, before us, informs us that we were not correct in our adjudication. M. Brierre de Boismont attributes the origination of this reform movement to a physician named Daquin, who forty years ago died at Chambery; and what Brierre de Boismont asserts is not to be lightly disregarded.

"The question of priority," remarks M. Brierre de Boismont, "is one of great importance in the interests of humanity, but instead of disputing thereon, the first thing to be done is to collate and compare the writings of both. Daquin published, in 1791, a work entitled, "A Medico-philosophical Treatise upon Insanity." In 1801, Pinel

* Annales Medico-Psychologiques. Janvier, 1854.

published his *Medico-philosophical Treatise on Mania*—an interval of ten years thus separating these authors.

Daquin asserted that it was possible to treat this disease by moral, rather than by physical agencies. One of the first reforms demanded by him, was the abolition of the dungeons in which the insane were then incarcerated. "Rare and strange animals," he observes, "are carefully tended in their cages, and supplied with all they require; the unfortunate lunatic is left in culpable neglect." He never ceased to insist upon the abandonment of chains, cells, &c., &c. He incessantly urged that the continued imprisonment in ill-ventilated cells, perpetuated or irrevocably fixed the mental disorder of those who had otherwise, by better treatment, been restored to reason. Daquin not only preached these reforms, but so far as lay in his power, he put them into practice; but unaided, he could in his day effect little, and that little again vanished after his death, until Pinel's benevolent and energetic mind carried on towards ultimate success the mighty change, with which his name must ever be associated.

Coercion, Daquin maintained, was only useful in the milder cases. "What is the use," he observes, "of having recourse to mechanical restraint when so celebrated a physician as Cullen avowed that he knew of no means of coercion that were at once easy and salutary." Daquin further urged improvemnt in diet, fresh air, exercise, occupation, as remedial measures of the first importance; and he enforced the advantages of mild behaviour and gentleness, mingled with firmness of deportment, towards the insane. Moral, rational treatment was the principle he never ceased to enforce. In all the points here referred to, are to be found the germs of the great reforms subsequently accomplished by Pinel, Esquirol, and others in this country.

Although the character of Pinel is granted by M. Brierre de Boismont to place him above suspicion of plagiarism, it seems improbable that Pinel should not have been aware of the writings of Daquin. At the same time, there is little doubt that similar views had originated in the mind of each, independently of any suggestions or information derived from the other. Daquin was the first to call attention to existing atrocities, and to attempt the reform required; he was the author of a method which has finally triumphed, but unaided, without hospital, pupils, without a press, or other auxiliary, he could do no more than enunciate doctrines which Pinel developed, by the help of all the advantages of one of the largest hospitals in Europe, with all the authority of the chief of a great school, with the advantages of intelligent aid, and at a time when the strongest disposition for a change of all established institutions affected the public mind. With all these favourable conditions, Pinel accurately interpreted and elaborated the noble sentiments of Daquin. The priority as to date rests indisputably with Daquin, but the more philosophic mind, the more energetic will, in a more propitious age, seized and improved opportunities which had not been granted by Providence to Daquin.

It is not credible, M. Brierre de Boismont urges, that such a work as that of Daquin could have been published in France, and have remained unknown to Pinel, who quotes so many other authors, ancient and contemporary. It is still further matter of astonishment that the same silence should have been maintained in his edition of 1809, in the *Clinique de Salpêtrière*, 1807, and in the six editions of the *Nosographie*, of which the last was published in 1818, although Daquin had dedicated his second edition to Pinel in 1804, five years before the publication of the second edition of Pinel's *Traité médico-philosophique sur l'alienation.*

"Can it be true," most pointedly asks M. Brierre de Boismont, "that there is in the heart of the most illustrious men a secret corner in which they hide the weaknesses of our nature; and must we pronounce, among these weaknesses, the impossibility of pronouncing the name of a rival, or, as it has been justly denominated by a modern author, *la conspiration du silence?* We withhold reply, but refer to the facts now related."

*Researches on Cretinism*, by M. Baillarger.—The author, in the first place, examines the definitions of Cretinism given by various writers, and compares these with the recorded descriptions, by different observers, of the condition to which the name *Cretinism* is applied.

Two principal opinions have been enunciated; the one consisting in the assimilation of this state with that of idiotcy, from which it is alleged to be separated only by certain accessory and unimportant characters; the second opinion regards Cretinism as a distinct malady.

M. Baillarger, in order to arrive at satisfactory conclusions on this question, has visited, during several months, the districts in which the malady prevails. The result of these researches, carried on in the Pyrenees and Alps, is, that Cretinism is to be defined as an "incomplete, irregular, and, most frequently, very tardy development of the organization;" and is, therefore, essentially different from endemic idiotcy.

*The Consequences of Epilepsy.*—The observations here referred to are extracted from a work in the press on Epilepsy, by M. Delasiauve. The consequences treated of, in this place, are those dependent on the general course of the disease, or its complications. These are enumerated, and described with reference to the primary malady, at the same time illustrated cases of the several complications are given. Among those which are noticed, are, apoplectic and inflammatory congestion, mania, stupidity, delirium, paralytic dementia, and idiotcy.

We quote parts of the author's observations on *stupidity* as a consequence or complication of epilepsy. It may be superfluous to state, that this is a mental condition frequently met with independently of epilepsy, presenting simply a constitutional dulness and slowness of ideas, weakness of memory, confusion in reasoning, and indecision of character.

In its higher degree, epileptic stupidity has all these defects in an exaggerated form approaching to the state of idiotcy. The intellects impaired, conversation impossible, from want of clearness of thought, and from inability of utterance. The patient obviously comprehending what is addressed to him, his countenance expresses all the intermediate gradations from dulness to stupefaction. This expression, observes M. Delasiauve, does not ordinarily indicate feelings of depression, but simply of suspension of cerebral action. It is intelligible how, under such circumstances, violence may occasionally be manifested, according as the patient is under the influence of sinister or automatic impulses. The intensity of this stupidity will vary with the progress of the original malady. With its subsidence, the intellectual powers may clear up and regain their former activity. This form of mental malady is more persistent than mania which results from epilepsy.

The following instance is given by the author:—

A youth was admitted into the Bicêtre, presenting the characters here described. He was a miller, had arrived from the provinces to be present at the festival of the 15th August in Paris. He was a mere automaton, could not tell whence he came, whither he was going, what his country was, where he then was, nor could he express any ailment. His countenance was inexpressive, as his mind was without thought, and although exhibiting a slight tinge of melancholy, he manifested no sentiment of chagrin or fear. In about eight days this chaos of the intellect seemed to clear up a little, after a succession of epileptic seizures.

M. Baillarger regards delirium tremens as connected with this form of stupidity, from its resemblance to the state of inebriety; the reaction giving rise to automatic impulses, which take their direction from sensorial illusions regarding surrounding objects. The hallucinations are generally of a sinister aspect, such as the supposed presence of assassins, robbers, spectres, &c.

M. Delasiauve further enters into a full consideration of the allied forms of mental derangement; as well as the other consequences of epilepsy above enumerated.

*Monomania, in relation to the Criminal Law.*—M. Victor Molinier has attempted in some degree to reconcile the discrepancy which so often occurs between legal and medical opinion upon the state of mind in criminals; a difference arising, according to M. Molinier, from the inquiry not having been made with a due regard to the respective domains of law and medicine; the latter, he urges, has merely to determine the fact of the existence of insanity—the former has to determine whether, at the time of the commission of a crime, the offender was, from his knowledge of right and wrong, responsible for his actions.

Herein, however, is the point of difficulty which M. Molinier has not quite cleared away.

Monomaniacs, M. Molinier urges, are aware when they commit a crime; they can make their election between the observance of the laws and the punishment of their violation; between the risk of the latter and the indulgence of an impulse originating in unrestrained passion or ill-regulated affections. The difference between the monomaniac and the culprit is only one of degree of moral depravity. To hold any other view, the author holds, is to destroy the free will and responsibility of man, and abolish the bulwarks and protection of society.

The doctrine here enunciated betrays rather the strong arm than the strong argument.

*Legal Medicine.*—In this department of the journal, M. Morel relates a case of feigned insanity, which was detected, and afterwards confessed by the culprit. Also, the history of a man who had violently torn out his wife's eyes, and concerning whose mental condition the opinion of M. Morel was required. This man had for many years been a good husband and a good father, industrious as a workman, and of excellent general character. Under the influence of derangement of his health he had become hypochondriacal, suspicious, and jealous of his wife; his delusions had been aggravated by illusions of the senses of sight and hearing—and actuated by these erroneous impression he had committed the violence already mentioned, with the intent at the moment to have committed actual murder.

In the *Therapeutic Report*, or extracts from other journals, we find statements of the beneficial effects of belladonna in neuralgic affections, by M. Sandras; of the successful employment of manganese in chlorotic affections, by M. Stoeber; of the serviceable administration of the fumes of nitrate of potash in asthma, by M. Trousseau; the mode of administration of the last-named remedy consisting in the saturation of paper in solution of the salt, and its ignition beneath the nostrils of the patient.

M. Recamier gives his statement of the success attending the use of cold affusion in puerperal convulsions. M. Aussaguel is here cited as disapproving of venesection in apoplexy. The advantages of the inhalation of chloroform in delirium tremens is quoted from Dr. Pratt's paper in the *American Journal.* A case of general paralysis, presenting intermittent characters, is reported as cured by sulphate of quinine.

*The Report of Proceedings of Learned Societies* is occupied with the discussion on monomania, at the *Société Médico-Psychologique,* May 30th, June 27th, July 25th, and October 30th.

In the *Review* department we find notices of the following works:—

"On Spirits, and their Fluid Manifestation," by M. le Marquis Eudes de M——; of which work the reviewer says—"I have read this book through from one end to the other, and my curiosity has not flagged for one instant. Its strange title is not a mere catch, as is too often seen on the covers of books. The text is as strange as the title. Not a shade of an artifice throughout. Here is *naïveté,* not wanting learning: moreover, here is a rare courage, the courage of self-opinion, which, bestowing a sort of heroic attitude upon an eccentric thought, elicits from the most hostile reader sympathy and respect. Not in our own age, beyond the strife of party, have we met with a writer who attacks with such aggressive serenity, or with a stronger faith in sarcasms, the scorn of what is called common sense. As if by a single blow to set at defiance all sneers and shrugs, the author has presented what he modestly terms his memoir to the Academy of Moral and Political Science, being the most competent tribunal, as, from the nature of its pursuits, it is less averse to separate the supernatural from sciences of observation."

The object which the Marquis has in view is, it appears, to demonstrate the presence and material intervention of spirits in the affairs of this world; and that their office is to worry poor humanity, and to augment its already numerous tribulations. These spirits, says the Marquis, will interfere either spontaneously, or by voluntary or involuntary invocation; spontaneously in certain mental or nervous affections, or, in the language of the author, "in hallucination and mysterious perceptions, in possession and prophetic voices, in mysterious neuropathics;" by voluntary invocation, in sorcery, magic, &c.; by involuntary invocation, in mesmerism, animal magnetism, spirit rappings, table-turning, &c.

The precise nature and characters of these fluidified spirits are not here recorded.

We need scarcely further to occupy our space with such speculations, which, indeed, to our common sense, would have been deemed to have been the production of the inmate of some asylum.

*Sauvons le Genre Humain!* such is the title of a work by M. Victor Hennequin; another exposition of *Fourierism!!*

The next publication brought under consideration is that of Dr. Hubert Valleroux, *Upon the Actual Condition of the Deaf Mute and the Blind.* Upon the nearest calculation, the author informs his readers, there are in France 27,286 deaf and dumb persons, or 1 in every 1356 inhabitants. This estimate M. Hubert believes to be below the truth. The various modes of instructing these, as well also the blind, are examined by M. Hubert, who proposes an entire new organization of the present system of education for these unfortunates. The most important feature of the scheme is that it proposes to educate the deaf and dumb and blind to agricultural pursuits, and, therefore, requires that the institutions for their benefit should be established in the country.

*The Hygiene of Body and Soul.*—M. Mar. Simon, known to us as the author of *Deontologie Médicale*, has published a work upon temperance, under the above title, and which, it appears, according to the reviewer, enforces religious motives also for the avoidance of the vice of drunkenness.

Notices of the Reports of Asylums, French and Foreign, conclude this portion of the journal.

---

